# Genetic diversity and population structure in five Inner Mongolia cashmere goat populations using whole-genome genotyping

**DOI:** 10.5713/ab.23.0424

**Published:** 2024-04-01

**Authors:** Tao Zhang, Zhiying Wang, Yaming Li, Bohan Zhou, Yifan Liu, Jinquan Li, Ruijun Wang, Qi Lv, Chun Li, Yanjun Zhang, Rui Su

**Affiliations:** 1 College of Animal Science, Inner Mongolia Agricultural University, Hohhot, Inner Mongolia Autonomous Region, 010018, China; 2 Grassland Research Institute, Chinese Academy of Agricultural Sciences, Hohhot, Inner Mongolia Autonomous Region, 010018, China; 3 Inner Mongolia Key Laboratory of Sheep and Goat Genetics Breeding and Reproduction, Hohhot, Inner Mongolia Autonomous Region, 010018, China; 4 Key Laboratory Of Mutton Sheep and Goat Genetics And Breeding, Ministry of Agriculture And Rural Affairs, Hohhot, Inner Mongolia Autonomous Region, 010018, China; 5 College of Animal Science and Technology, Inner Mongolia Minzu University, Tongliao, Inner Mongolia Autonomous Region, 028000, China

**Keywords:** Cashmere Goat, Genetic Diversity, Inner Mongolia Autonomous Region, Population Structure

## Abstract

**Objective:**

As a charismatic species, cashmere goats have rich genetic resources. In the Inner Mongolia Autonomous Region, there are three cashmere goat varieties named and approved by the state. These goats are renowned for their high cashmere production and superior cashmere quality. Therefore, it is vitally important to protect their genetic resources as they will serve as breeding material for developing new varieties in the future.

**Methods:**

Three breeds including Inner Mongolia cashmere goats (IMCG), Hanshan White cashmere goats (HS), and Ujimqin white cashmere goats (WZMQ) were studied. IMCG were of three types: Aerbas (AEBS), Erlangshan (ELS), and Alashan (ALS). Nine DNA samples were collected for each population, and they were genomically re-sequenced to obtain high-depth data. The genetic diversity parameters of each population were estimated to determine selection intensity. Principal component analysis, phylogenetic tree construction and genetic differentiation parameter estimation were performed to determine genetic relationships among populations.

**Results:**

Samples from the 45 individuals from the five goat populations were sequenced, and 30,601,671 raw single nucleotide polymorphisms (SNPs) obtained. Then, variant calling was conducted using the reference genome, and 17,214,526 SNPs were retained after quality control. Individual sequencing depth of individuals ranged from 21.13× to 46.18×, with an average of 28.5×. In the AEBS, locus polymorphism (79.28) and expected heterozygosity (0.2554) proportions were the lowest, and the homologous consistency ratio (0.1021) and average inbreeding coefficient (0.1348) were the highest, indicating that this population had strong selection intensity. Conversely, ALS and WZMQ selection intensity was relatively low. Genetic distance between HS and the other four populations was relatively high, and genetic exchange existed among the other four populations.

**Conclusion:**

The Inner Mongolia cashmere goat (AEBS type) population has a relatively high selection intensity and a low genetic diversity. The IMCG (ALS type) and WZMQ populations had relatively low selection intensity and high genetic diversity. The genetic distance between HS and the other four populations was relatively high, with a moderate degree of differentiation. Overall, these genetic variations provide a solid foundation for resource identification of Inner Mongolia Autonomous Region cashmere goats in the future.

## INTRODUCTION

Archaeological and genetic evolutionary evidence suggests that goats were domesticated in the early Neolithic period (10,000 years ago), and were the first domesticated ruminants [1,2]. Goats are mainly distributed in developing and underdeveloped countries, such as Mongolia, Iran, India, and Afghanistan, and including China, which has abundant goat genetic resources. China is ranked first in the number of cashmere goats raised, production, quality and cashmere trade [3,4]. Regions with relatively high cashmere production are concentrated in Inner Mongolia, Liaoning, Xinjiang, Qinghai, Gansu, Henan, and Tibet. Inner Mongolia cashmere goats are well-known for their high cashmere production and quality, and large body size. They are the largest Chinese cashmere goat population and have played a crucial role in goat breeding in the country. Additionally, cashmere goats are key protected animals in China and are strictly prohibited from export. Inner Mongolia has three cashmere goat breeds named by the National Genetic Resources Commission including Inner Mongolia, Hanshan White and Ujimqin White. Among them, there are three types of Inner Mongolia cashmere goats including: Aerbas (AEBS), Erlangshan (ELS), and Alashan (ALS) [5,6]. As a charismatic species, goats play an important role in the animal husbandry economy, especially in remote mountain areas. Due to a lack of genetic resource conservation, the genetic diversity of many goat breeds is continuously declining. Goat genetic resource diversity is related to national ecological and agricultural security. Protecting cashmere goat genetic resources and ensuring population genetic diversity will help protect special traits, maintain working cashmere goat breeds and promote sustainable development of the modern cashmere goat industry [7].

Genetic diversity generally refers to the sum of genetic variation of different individuals within a breed, usually with single nucleotide polymorphism (SNP) as the minimum unit [8]. At the earliest stage, genetic markers are obtained by restricting fragment length polymorphisms and microsatellite polymorphisms [9]. For now, SNP markers are the most common in the genome, characterized by large number and high density. More than 80% of genome genetic variation is caused by SNPs. Numerous studies have analyzed genetic diversity and population structure based on whole genome re-sequencing or SNPs chip data [10–12].

Although many studies on species genetic diversity and conservation of genetic resources have been conducted, there are relatively few on goat genetic diversity based on high-depth whole genome re-sequencing data, and none on Inner Mongolia cashmere goats. Therefore, for the first time, we determine the genetic diversity and population structure among native goat breeds in the Inner Mongolia Autonomous Region using whole-genome re-sequencing genotyping. This is vitally important for protecting the local cashmere goat populations.

## MATERIALS AND METHODS

### Sample collection

DNA samples were obtained from five populations of Inner Mongolia cashmere goats lodged in the sheep&goats Genetic Resource Bank of the Inner Mongolia Agricultural University, including Inner Mongolia cashmere goats (Aerbas type, AEBS; Erlangshan type, ELS; Alashan type, ALS), Hanshan white cashmere goats (HS) and Ujimqin white cashmere goats (WZMQ). The samples were from nine adult does in each population, all of them from the original breeding farm (Table 1). All DNA samples were measured for concentrations and optical density (OD) values using a nucleic acid protein analyzer. Concentrations were >50 ng/μL and OD values between 1.6 and 1.9, so all samples were used in subsequent analyses.

### Whole genome resequencing, quality control and single nucleotide polymorphism calling

DNBSEQ-T7 PE150 was used to construct sequencing libraries for each genomic DNA sample. Reads quality obtained by re-sequencing was controlled. Reads containing adaptor sequences and with N base content >10% were removed. Additionally, when the number of bases with a quality value <5 in any read exceeded 50% of the whole read, the paired reads were removed. BWA (Burrows-Wheeler Alignment) software (parameter MEM-T4-K32-m) was used to align the clean reads to the assembled high-quality reference genome in our laboratory (Youngmok and Dongsu, in prep.). Replicate reads were removed using rmbup commands in Samtools software. The Samtools mpileup module (-q 1 -C 50 -t SP-t DP-m 2) was used to load all sample alignment files, and then the bcftools (-mv-f GQ) call module was used to resolve [13]. 30,601,671 raw SNPs were obtained. The vcfutils varFilter module (-Q 20-d 2-D 100000) was used for quality control, and the original mutation sites of each sample were finally obtained. SNPs with 7<depth≤138, miss<0.1 and maf>0.05 were filtered and screened, providing about 17,214,526 high-quality SNPs (Table 2). Then, ANNOVAR software was used to perform functional annotation on the detected gene variants [14].

### Genetic diversity analysis

The stacks software population module was used to analyze the expected heterozygosity (H_e_), observed heterozygosity (H_o_) and proportion of polymorphic sites (Pn%) of the filtered high-quality SNPs [15]. The Plink software genome method was used to obtain the ratio of Identity by Descent (PI_HAT) [16]. The SNPs average inbreeding coefficient (F) was performed using a python script (F = 1–H_o_/H_e_).

### Principal component analysis, phylogenetic tree construction and population structure analysis

The GCTA software gcta64 --grm cashmeregoats_grm --pca 3 --out cashmeregoats_pca command was used to perform principal component analysis (PCA) of SNPs for the five populations [17]. The three largest eigenvectors generated by PCA analysis were taken as the principal axes, and the R plot function was used to draw PCA plots [18]. The population genetic distance of the filtered high-quality SNPs was calculated, and then the TreeBest software NJ method was used to construct the SNPs phylogenetic tree. Finally, the population phylogenetic tree was drawn on the itol website ( https://itol.embl.de/). The admixture software admixture method was used to analyze the SNPs population structure [19], and the barplot function in R software was used to plot the SNPs lineage composition.

### Linkage disequilibrium decay analysis

Linkage disequilibrium (LD) decay was analyzed using the PopLDdecay (version 3.40) software PopLDdecay method, and the LD decay map was plotted using the Plot_MultiPop method [20].

### Population genetic differentiation

Different sub-populations are affected by environments or by artificial selection, resulting in an increase or decrease in genotype frequency of certain loci. Therefore, at this locus, the magnitude of the difference between sub-populations is higher than that between normal sub-populations. Significantly different SNPs are considered to have been selected. Generally, *F_st_* is used to indicate the size of the difference between sub-populations, with higher *F_st_* indicating greater sub-population differences. We used vcftools software to calculate *F_st_* between sub-populations [21], and also to determine *θ*_π_ which indicates population nucleotide diversity fractions on SNPs. Usually, a lower polymorphism indicates a higher degree of selection.

## RESULTS

### Whole genome resequencing

After library construction and sequencing with DNBSEQ-T7 PE150, raw data were produced for each individual (Supplementary Table S1). The sequencing data of 45 individuals in the five goat populations showed that Q20 and Q30 were >98% and ≥93%, respectively. Guanine and cytosine (GC) content was between 42% and 44%, which indicated that sample quality was good and samples could be used for subsequent analysis. The re-sequencing data of the five populations were compared with the reference genome. The mapping rate was between 96.83% to 99.95%, average sequencing depth was 28.5×, and range of individual sequencing depth was 21.13× to 46.18× (Supplementary Table S2). The number of raw SNPs was about 30,601,671, and after filtering and screening, 17,214,526 high-quality SNPs were finally obtained.

SNP call and annotation showed that 91,647 SNPs were found in the 1 kb region upstream of the genes, and 194,016 SNPs were found in the exon region, including 670 stop gain, 123 stop loss, 62,948 synonymous mutations, and 46,878 non synonymous mutations. The intron region contained 4,859,122 SNPs, and the gene interval 11,970,858 SNPs. The downstream 1 kb region contained 94,018 SNPs and 232 SNPs at the splice site mutation (Supplementary Table S1).

### Genetic diversity analysis

Genetic diversity parameters were estimated using filtered high quality SNPs (Table 3). Proportion of polymorphic loci (*P_N_*) values of the five populations ranged from 79.28% to 88.57%, with the largest in the ALS population (*P_N_* = 88.57) and the smallest in the AEBS population (*P_N_* = 79.28). The AEBS population inbreeding coefficient was relatively high (0.1348), but for the WZMQ, ELS, and ALS populations it was low (F_is_ from 0.0731 to 0.0773). The HS inbreeding coefficient was the highest among the five populations. Overall, selection intensity in AEBS was relatively high, while in HS it was low. PI_HAT reflects the degree of genetic relationship between two individuals. Among the five populations, the highest and lowest PI_HAT were 0.1021 and 0.010, for the AEBS and ALS populations, respectively. MAF of these five populations ranged from 0.1859 to 0.1941. The observed heterozygosity (H_o_) and expected heterozygosity (H_e_) of the five populations were relatively low, ranging from 0.2755 to 0.2886, and from 0.2554 to 0.2721, respectively. Observed heterozygosity was highest in ELS, and lowest in HS, while expected heterozygosity, was lowest in AEBS, and highest in ALS.

### Principal component analysis, phylogenetic tree and population structure

**Figure 1:**
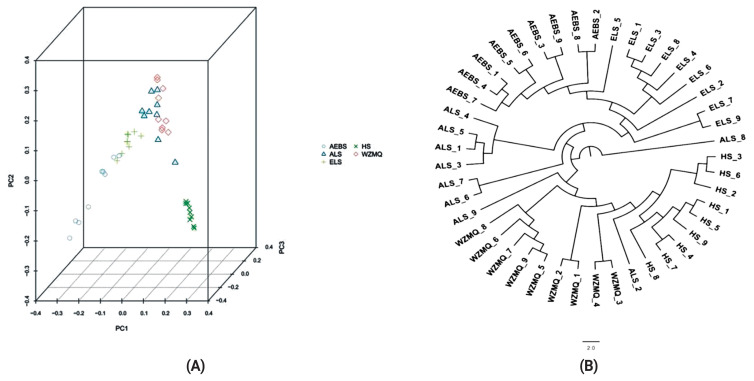
(A) Multidimensional scaling plot depicting the relationships among the five populations. PC1, first component; PC2, second component. (B) Phylogenetic tree.

Principal component analysis indicated that the genetic distance between HS and the other four populations was relatively high, and it was clearly separated from other populations. The genetic distances between ALS and WZMQ, and AEBS and ELS were relatively close (Figure 1A). The phylogenetic tree showed that the five populations could be divided into two branches, with the ALS population closest to the root. The HS and AEBS populations were separated based on the WZMQ and ELS populations, respectively (Figure 1B).

**Figure 2:**
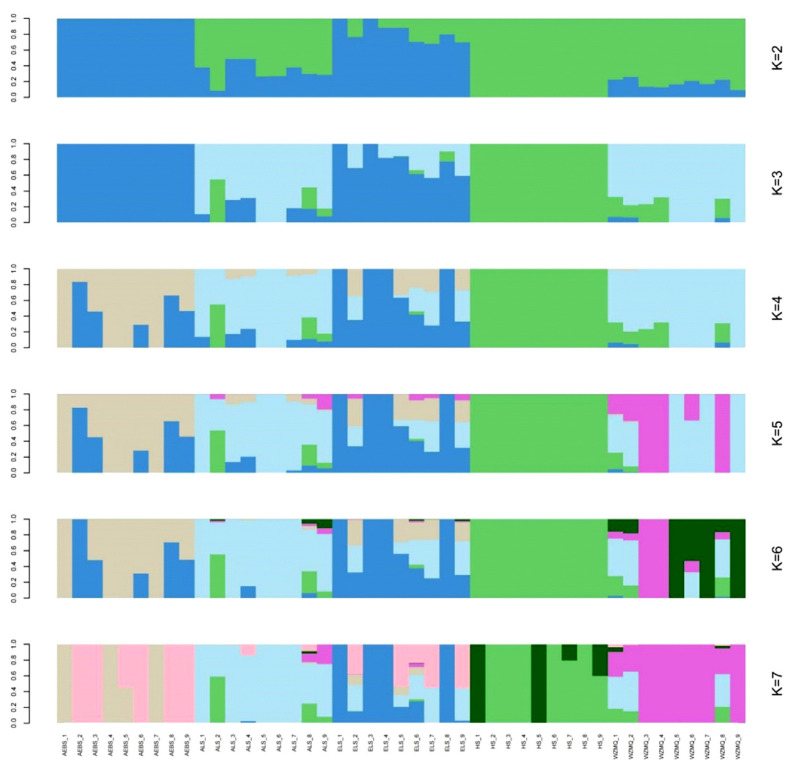
Admixture analysis of cashmere goat populations for a range of K-values (2 to 7).

Population structure was used to analyze genetic differences between sub-populations (Figure 2). There was an increasing trend of cross-validation error values according to k-values, and cross-validation error values ranged from 0.5572 to 0.9042. The maximum likelihood value was 2, indicating that the five cashmere goat populations should be divided into two subgroups. When K = 2 and K = 3, the AEBS and HS populations were relatively independent, and there was a certain degree of genetic hybridization among the ALS, ELS, and WZMQ populations. When K = 4, 5, and 6, the HS group was relatively independent, while other populations had a certain degree of confusion. Overall, the five goat populations in the Inner Mongolia Autonomous Region should be divided into two subpopulations, consistent with the principal components and phylogenetic tree analyses.

### Estimation of population genetic differentiation parameters

**Figure 3:**
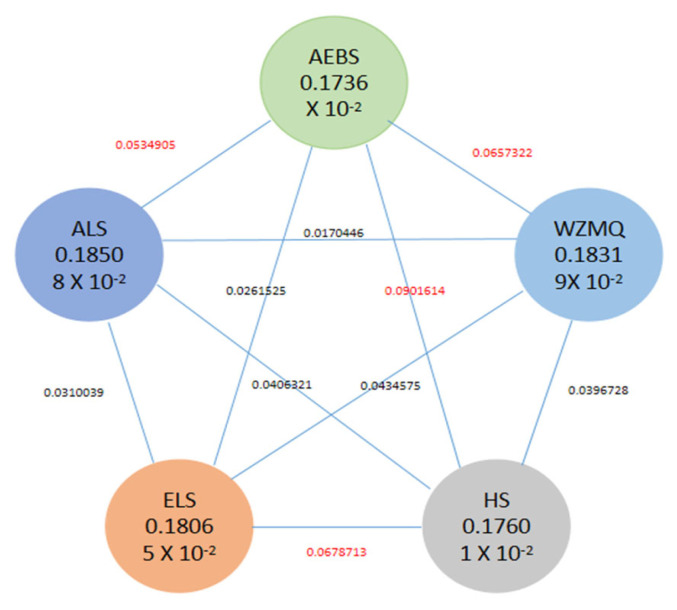
Population genetic differentiation parameters and nucleotide diversity. F_st_, the connection value between two groups; *θ*_π_, the corresponding circle within the population.

Variations in the genetic differentiation parameter (*F_st_*) represent the magnitude of difference between two populations (0 to 1), where the larger the *F_st_* value, the greater the difference. A moderate degree of genetic differentiation was found between HS and ELS, HS and AEBS, AEBS and ALS, and AEBS and WZMQ. The degree of differentiation between AEBS and HS was relatively high, while between the other populations was relatively low and not obvious (Figure 3).

The nucleotide polymorphism value (*θ*_π_) represents the degree of nucleotide polymorphism among individuals in the same population, and the higher the value of *θ*_π_, the greater the polymorphism. Nucleotide diversity values of the five populations from high to low were ALS>WZMQ>ELS> HS>AEBS, but there was no significant difference in genetic diversity among the five populations (Figure 3).

**Figure 4:**
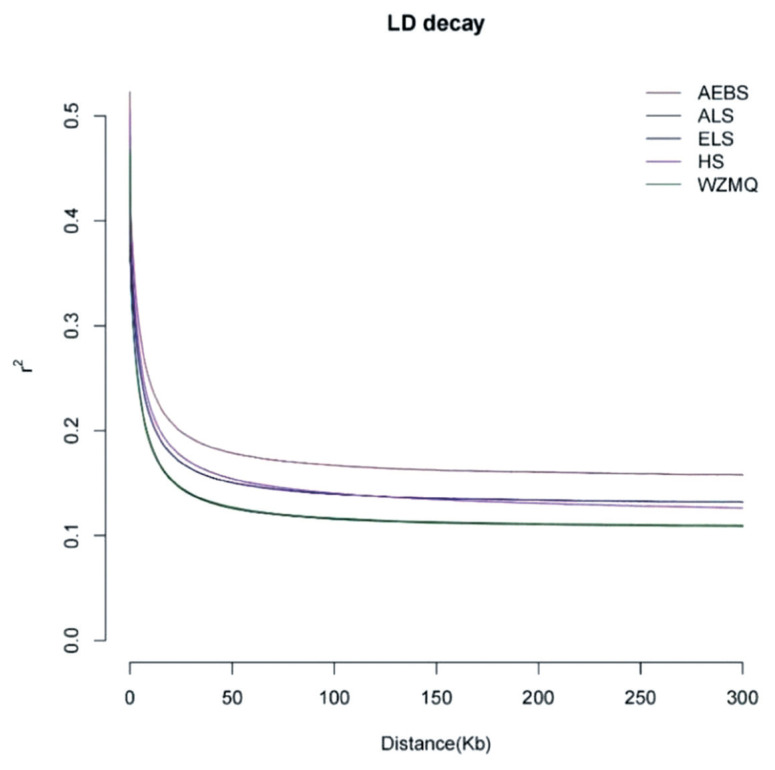
Genome-wide average LD decay estimated for five Inner Mongolia cashmere goat populations. The vertical axis denotes the LD coefficient r^2^ and the horizontal axis denotes the distance between the genes. LD, linkage disequilibrium.

We analyzed the degree of LD of SNPs within 300 kb among the five cashmere goat populations. The LD had a downward trend with increasing physical distance between SNPs (Figure 4; Table 4). Within a physical distance of 1 to 50 kb, LD decay degree from high to low was AEBS>HS> ELS>WZMQ/ALS. After 150 kb, the declining degree of LD ranges from high to low was AEBS>ELS>HS>WZMQ/ALS. Within a range of 1 to 300 kb, the AEBS population consistently maintained the highest LD and had the slowest decay rate, indicating low population genetic diversity. The difference in LD between ELS and HS was not significant. In the range of 1 to 50 kb, the decay rate of ELS was slightly higher than the HS population, and the ELS LD value was relatively low, indicating that its population genetic diversity was slightly enriched. ALS and WZMQ LD values and decay rates were almost identical over the entire distance range, both maintaining the fastest decay rate and the lowest LD, indicating the richest genetic diversity and the least artificial selection.

## DISCUSSION

Genetic diversity parameter estimates indicated that proportion of polymorphism and expected heterozygosity were lowest in the AEBS population, and the proportion of homology and average inbreeding coefficient were the highest. This may be due to the relatively large selection intensity for this population. A breeding program for this population began in 1998, and production performance has significantly increased. The average AEBS population inbreeding coefficient was 0.1348, indicating that a certain proportion of inbreeding occurred within three generations. In the ALS and WZMQ populations, the proportion of polymorphism and expected heterozygosity were relatively high, and the proportion of homology and average inbreeding coefficient were relatively low, indicating that both populations were under relatively low selection intensity. Furthermore, the average inbreeding coefficient was only 0.07, indicating that the proportion of inbreeding within three generations was very low. As a special population, the proportion of polymorphism in the HS population was high, indicating that it was not under selection, and that the selection scheme needs to be further improved. Generally, degree of heterozygosity is positively correlated with population diversity, with higher heterozygosity indicating richer population diversity [22]. If the average population heterozygosity is >0.5, it indicates that the population has rich genetic diversity [23]. The observed heterozygosity of the five populations estimated in this study was not significantly different, ranging from 0.2755 to 0.2886. Zhao et al [24] used microsatellite markers to estimate the expected and observed heterozygosity of four Inner Mongolia cashmere goat populations and found that estimated values were much higher than in this study. This may be related to variables such as methods, sample collection time and sample size.

The HS population and the other four populations were significantly separated. However, the genetic distances between WZMQ, AEBS, ALS, and ELS were very close, indicating that genetic exchange was low [25], as confirmed by the population differentiation index. When the *F_st_* value is <0.05, it indicates a small genetic differentiation between populations, while from 0.05 to 0.15 it indicates moderate differentiation [26]. The *F_st_* values between any two Inner Mongolia cashmere goat populations were relatively low, ranging from 0.017 to 0.090, indicating small or moderate differentiation. This may be due to long-term closed breeding and continuous selection in the Inner Mongolia cashmere goat population, resulting in relatively homozygous genes. Nonetheless, the Inner Mongolia cashmere goat population has been subjected to genetic resource protection and prevented from hybridizing with other populations.

The AEBS and ALS populations on the phylogenetic tree were clustered into one category, as were the HS and WZMQ populations, while the ALS population intersected these two major categories. This may be explained by geographical factors. Additionally, population structure analysis showed that K = 2 when the CV value was lowest, indicating that the five cashmere goat populations were divided into two subgroups, which is consistent with PCA and phylogenetic tree results. Therefore, when K = 2, the HS population was independent, and the other four populations had different degrees of genetic intercourse flow.

Decay rate represents population genetic diversity, the faster the decay rate, the richer the population diversity [27–30]. The AEBS population LD decay rate was the slowest, indicating that its genetic diversity was the lowest, which was consistent with the previous genetic diversity parameter estimation results, and might be related to continuous artificial and closed breeding. The WZMQ and ALS populations had the fastest LD decay rate and higher genetic diversity, which was also confirmed by the population differentiation index.

The population nucleotide diversity value (*θ*_π_) also reflects genetic diversity, the larger the *θ*_π_ value, the higher the population diversity. The ALS and WZMQ populations had higher genetic diversity, as indicated by *θ*_π_ values of 0.0019 and 0.0018. The AEBS population *θ*_π_ value was the lowest, a result consistent with the LD decay map.

## CONCLUSION

The estimated genetic diversity parameters of five cashmere goat populations in the Inner Mongolia Autonomous Region showed that the AEBS population had the lowest polymorphic rate and expected heterozygosity, while the average coefficient of kinship and average inbreeding coefficient were the highest, indicating that this population has a high selection intensity and a certain proportion of inbreeding within three generations. Compared to other populations, the ALS and WZMQ polymorphic rate and expected heterozygosity were high, while the average coefficient of kinship and average inbreeding coefficient were relatively low, indicating relatively low selection intensity. Principal component analysis and phylogenetic tree results showed that the genetic distance between HS population and the other four populations was relatively high. Moreover, degree of differentiation between HS and other populations was relatively high, indicating a moderate degree of genetic differentiation. Among them, the HS and AEBS populations had the highest degree of differentiation, while among the AEBS, ELS, and ALS populations, it was relatively low. Linkage disequilibrium decay results showed that the LD decay trend of the five populations (AEBS, ELS, ALS, HS, and WAMQ) was basically consistent with increased distance between SNPs. The AEBS and HS population decay degree rates were relatively low, indicating relatively low genetic diversity and probably frequent artificial breeding.

## Figures and Tables

**Table 1:** Characteristics of the Inner Mongolia cashmere goats sampled from different populations

Name	Sex	No.	Stock
AEBS	Female	9	Inner Mongolia Yiwei White Cashmere Goat Co., Ltd
ELS	Female	9	Inner Mongolia Mizhen International Trade Co., Ltd
ALS	Female	9	Alxa Left Banner Goat Breeding Farm
HS	Female	9	Chifeng Bahrain Right Banner Hanshan Goat Breeding Farm
WZMQ	Female	9	Ujimqin White Goat Original Breeding Farm

AEBS, Inner Mongolia cashmere goats (Aerbas type); ELS, Inner Mongolia cashmere goats (Erlangshan type); ALS, Inner Mongolia cashmere goats (Alashan type); HS, Hanshan white cashmere goats; WZMQ, Ujimqin white cashmere goats.

**Table 2:** Single nucleotide polymorphism annotation results of five Inner Mongolia cashmere goat populations

Category	Number of SNPs
Upstream	91,647
Exonic	
UTR3	70,383
UTR5	12,741
UTR5;UTR3	17
Stop gain	670
Stop loss	123
Synonymous	62,948
Non-synonymous	46,878
Unknown	256
Intronic	4,859,122
Splicing	232
Downstream	94,018
Upstream/downstream	4,633
Intergenic	11,970,858
ts	12,126,852
tv	5,087,674
ts/tv	2.383
Total	17,214,526

SNPs, single nucleotide polymorphisms; UTR, untranslated region; upstream/downstream, the upstream of a gene and downstream of another gene; ts, transitions; tv, transversions; ts/tv, the ratio of transitions to transversions.

**Table 3:** Estimates of genetic diversity parameters of five Inner Mongolia cashmere goat populations

Breed	Number	P_N_	PI_HAT	MAF	H_e_	H_o_	F_is_
AEBS	9	79.28	0.1021	0.1859	0.2554	0.2806	0.1348
ELS	9	85.50	0.0702	0.1915	0.2660	0.2886	0.0773
ALS	9	88.57	0.0109	0.1941	0.2721	0.2842	0.0731
HS	9	80.98	0.0398	0.1878	0.2590	0.2755	0.1404
WZMQ	9	87.02	0.0411	0.1928	0.2697	0.2878	0.0737

P_N_, proportion of polymorphic marker; PI_HAT, the proportion of identity by descent; MAF, minor allele frequency; H_e_, expected heterozygosity values; H_o_, observed heterozygosity values; F_is_, inbreeding coefficients; AEBS, Inner Mongolia cashmere goats (Aerbas type); ELS, Inner Mongolia cashmere goats (Erlangshan type); ALS, Inner Mongolia cashmere goats (Alashan type); HS, Hanshan white cashmere goats; WZMQ, Ujimqin white cashmere goats.

**Table 4:** Linkage imbalance decay trend

Distance class (kb)	ALS	AEBS	HS	WZMQ	ELS
0–20	0.423	0.484	0.467	0.429	0.446
20–40	0.377	0.438	0.421	0.382	0.400
40–60	0.367	0.429	0.411	0.373	0.390
60–80	0.363	0.425	0.406	0.369	0.387
80–100	0.362	0.424	0.405	0.367	0.386
100–120	0.362	0.424	0.405	0.367	0.386
120–140	0.362	0.424	0.405	0.367	0.386
140–160	0.362	0.423	0.405	0.366	0.385

ALS, Inner Mongolia cashmere goats (Alxa type); AEBS, Inner Mongolia cashmere goats (Aerbas type); HS, Hanshan white cashmere goats; WZMQ, Ujimqin white cashmere goats; ELS, Inner Mongolia cashmere goats (Erlangshan type).

## SUPPLEMENTARY MATERIAL

**Supplementary Table S1**. Whole genome resequencing data quality in five Inner Mongolia cashmere goats populations

**Supplementary Table S2**. Sequencing depth and coverage
