# Supplementary material for: Genetic diversity and population structure in five Inner Mongolia cashmere goat populations using whole-genome genotyping
Source: Anim Biosci. 2024 Apr 1;37(7):1168–76. doi: 10.5713/ab.23.0424 (PMC11222833; doi:10.5713/ab.23.0424)
Supplement: Supplementary file 2 [file ab-23-0424-Supplementary-Table-2.pdf]

WZMQ_5	98.96;98.07	96.44;93.79	43.28;43.20	1,053,138,620	157,970,793,000
WZMQ_6	98.85;97.59	96.02;92.28	43.19;43.11	315,350,484	47,302,572,600
WZMQ_7	98.85;97.51	95.98;91.99	43.20;43.11	841,273,826	126,191,073,900
WZMQ_8	99.02;97.91	96.49;93.03	43.35;43.27	980,141,220	147,021,183,000
WZMQ_9	98.18;97.86	94.40;92.92	43.67;43.64	411,045,930	61,656,889,500

Table.S2 Sequencing depth and coverage

Sample	Clean reads	Clean base	Mapped_read	Mapping rate	Average depth	Coverage 1X	Coverage 4X
AEBS_ 1	470012766	70501914900	469683220	99.93%	24.51	97.78%	96.96%
AEBS_2	520964324	78144648600	519988246	99.81%	27.03	97.81%	97.02%
AEBS_3	602003082	90300462300	600840773	99.81%	31.26	97.82%	97.21%
AEBS_4	707317524	1.06098E+11	706607661	99.90%	36.82	97.45%	96.85%
AEBS_5	830256536	1.24538E+11	829409482	99.90%	43.40	97.41%	96.88%
AEBS_6	888858980	1.33329E+11	887900911	99.89%	46.18	97.49%	97.04%
AEBS_7	763292028	1. 14494E+11	762530928	99.90%	39.95	97.38%	96.80%
AEBS_8	542919614	81437942100	542353533	99.90%	28.24	97.56%	97.07%
AEBS_9	702670266	1.05401E+11	701855869	99.88%	36.72	97.48%	96.93%
ALS_ 1	484520948	72678142200	484144736	99.92%	25.25	97.65%	96.56%
ALS_2	531675250	79751287500	531161317	99.90%	27.77	97.80%	97.05%
ALS_3	456686546	68502981900	442224404	96.83%	23.27	97.54%	96.33%
ALS_4	472519716	70877957400	472029547	99.90%	24.77	97.72%	96.84%
ALS_5	511511402	76726710300	511005549	99.90%	26.98	97. 11%	96. 13%
ALS_6	464967900	69745185000	464374652	99.87%	24.46	97. 16%	96. 16%
ALS_7	602753886	90413082900	602090492	99.89%	31.64	97.38%	96.75%
ALS_8	579852510	86977876500	579198314	99.89%	30.51	97.28%	96.53%
ALS_9	500879838	75131975700	500564969	99.94%	26.36	97.31%	96.49%
ELS_ 1	460121700	69018255000	459862250	99.94%	24.06	97.78%	96.94%
ELS_2	398225698	59733854700	397991794	99.94%	20.82	97.83%	96.89%
ELS_3	422205580	63330837000	421947934	99.94%	22.13	97.73%	96.74%
ELS_4	487330324	73099548600	487075461	99.95%	25.55	97.74%	96.82%
ELS_5	457212522	68581878300	456913417	99.93%	24.04	97.33%	96.47%
ELS_6	446318986	66947847900	446081336	99.95%	23.56	97.20%	96. 13%
ELS_7	446675018	67001252700	446420578	99.94%	23.56	97. 17%	96.09%
ELS_8	459664312	68949646800	459389902	99.94%	24.10	97.30%	96.47%
ELS_9	467035178	70055276700	466777328	99.94%	24.54	97.34%	96.52%
HS_1	544421474	81663221100	543978530	99.92%	28.57	97.53%	96.58%
HS_2	475405336	71310800400	475033861	99.92%	24.89	97.58%	96.64%
HS_3	564044856	84606728400	563674706	99.93%	29.51	97.74%	96.96%
HS_4	521978040	78296706000	521606940	99.93%	27.41	97.61%	96.67%
HS_5	514756418	77213462700	514431888	99.94%	27.15	97. 17%	96.21%
HS_6	529415502	79412325300	529108301	99.94%	27.86	97.25%	96.41%
HS_7	615547596	92332139400	615124572	99.93%	32.36	97. 17%	96.32%
HS_8	507492742	76123911300	507124498	99.93%	26.70	97.27%	96.34%
HS_9	535479854	80321978100	535131916	99.94%	28.20	97. 18%	96. 19%
WZMQ_	474481366	71172204900	473960340	99.89%	24.80	97.73%	96.85%
WZMQ_	561040632	84156094800	560659378	99.93%	29.35	97.66%	96.80%
WZMQ_	453374214	68006132100	452801987	99.87%	23.74	97.63%	96.68%

WZMQ_	624179968	93626995200	623722436	99.93%	32.49	97.79%	97.08%
WZMQ_	601486324	90222948600	601024893	99.92%	31.54	97.26%	96.55%
WZMQ_	553621666	83043249900	553209784	99.93%	29.08	97.22%	96.42%
WZMQ_	621742500	93261375000	621305917	99.93%	32.62	97.36%	96.58%
WZMQ_	564060616	84609092400	563628500	99.92%	29.55	97.40%	96.63%
WZMQ_	632165318	94824797700	631659501	99.92%	33.03	97.34%	96.61%
